# What happens in the brain?

**DOI:** 10.7554/eLife.82258

**Published:** 2022-09-27

**Authors:** Faye Smith, Timothy D Griffiths

**Affiliations:** 1 School of Education, Communication and Language Sciences, Newcastle University Newcastle upon Tyne United Kingdom; 2 Biosciences Institute, Newcastle University Newcastle upon Tyne United Kingdom

**Keywords:** developmental language disorder, qMRI, microstructure, striatum, myelin, Human

## Abstract

A new imaging method reveals previously undetected structural differences that may contribute to developmental language disorder.

**Related research article** Krishnan S, Cler GJ, Smith HJ, Willis HE, Asaridou SS, Healy MP, Papp D, Watkins KE. 2022. Quantitative MRI reveals differences in striatal myelin in children with DLD. *eLife* **11**:e74242. doi: 10.7554/eLife.74242.

Approximately 10% of children have problems understanding or using spoken language. Given the critical role that language has in engaging with society and learning at school, these problems have profound consequences for the individuals affected. Language difficulties can be associated with hearing loss, a general learning disability, or social disadvantages. However, children can still develop a language disorder in the absence of these factors, suggesting that differences in brain structure or function may play a role.

A variety of changes to the brain may cause a developmental language disorder (DLD). For example, it has been suggested that individuals with DLD have a general problem with processing complex sounds that change rapidly over time, including speech (Tallal et al., 1996). In this instance, we might expect a cause of DLD to reside in the auditory part of the brain. DLD may also specifically impact how speech sounds are processed or the brain’s ability to remember these sounds over a short period of time. If this is the case, the language centres on the left side of the brain or the communications between them would be expected to be abnormal. The severe grammatical difficulties experienced by children with DLD suggests that the language centre Broca’s area, which is involved in forming complex sentences, is likely to play a role in the disorder.

Deep structures in the centre of the brain known as the basal ganglia have also been implicated in DLD. The basal ganglia help us to acquire the motor skills needed to perform certain tasks, such as riding a bike or typing on a computer. This type of procedural learning is also required to learn sequences of heard or spoken speech sounds. Defects in this basal ganglia system have been the basis for theories that seek to explain the cause of DLD (Krishnan et al., 2016; Ullman et al., 2020).

Previous studies have used magnetic resonance imaging (MRI) to study the brain anatomy of children with DLD. While standard MRI scanning in hospitals can pick up the abnormal folding of the cerebral cortex seen in some cases of DLD (Guerreiro et al., 2002), it has not detected structural abnormalities predicted to occur in the language centres and basal ganglia of children with DLD. Now, in eLife, Saloni Krishnan from the University of Oxford and Royal Holloway, University of London and colleagues report how they used a new MRI technique to define neural differences in DLD (Krishnan et al., 2022).

A previous study based on structural scans of the brain revealed that children with DLD had less white matter (which transfers information between neural areas) on the left side of the brain between the motor and language areas (Jäncke et al., 2007). Later studies used an approach called diffusion tension imaging (DTI) to examine nerve fibre tracts in the left side of the brain. This revealed abnormal connections between the areas of the brain responsible for language (Lee et al., 2020a), as well as connections between these areas and the basal ganglia (Lee et al., 2020b).

Krishnan and colleagues (who are based in the United Kingdom, United States and Canada) measured a signal called the magnetization transfer saturation (MTsat), which quantifies the amount of myelin covering nerves. Myelin is an insulating material that increases the speed at which electrical impulses travel down nerve fibres. Rather than examining the amount of myelin in the long fibre tracts in white matter between brain areas, MTsat examines local concentrations of myelin in grey matter areas that process speech information. In effect, it examines a feature of local brain circuits rather than the long-range connections between them.

Krishnan et al. applied this approach to brain images from 89 children, 33 of whom had DLD. This revealed that children with DLD had local decreases in myelin in the left striatum within the basal ganglia and Broca’s area (Figure 1). These structural differences provide further evidence that the basal ganglia and language centre have a key role in DLD.

**Figure 1.**
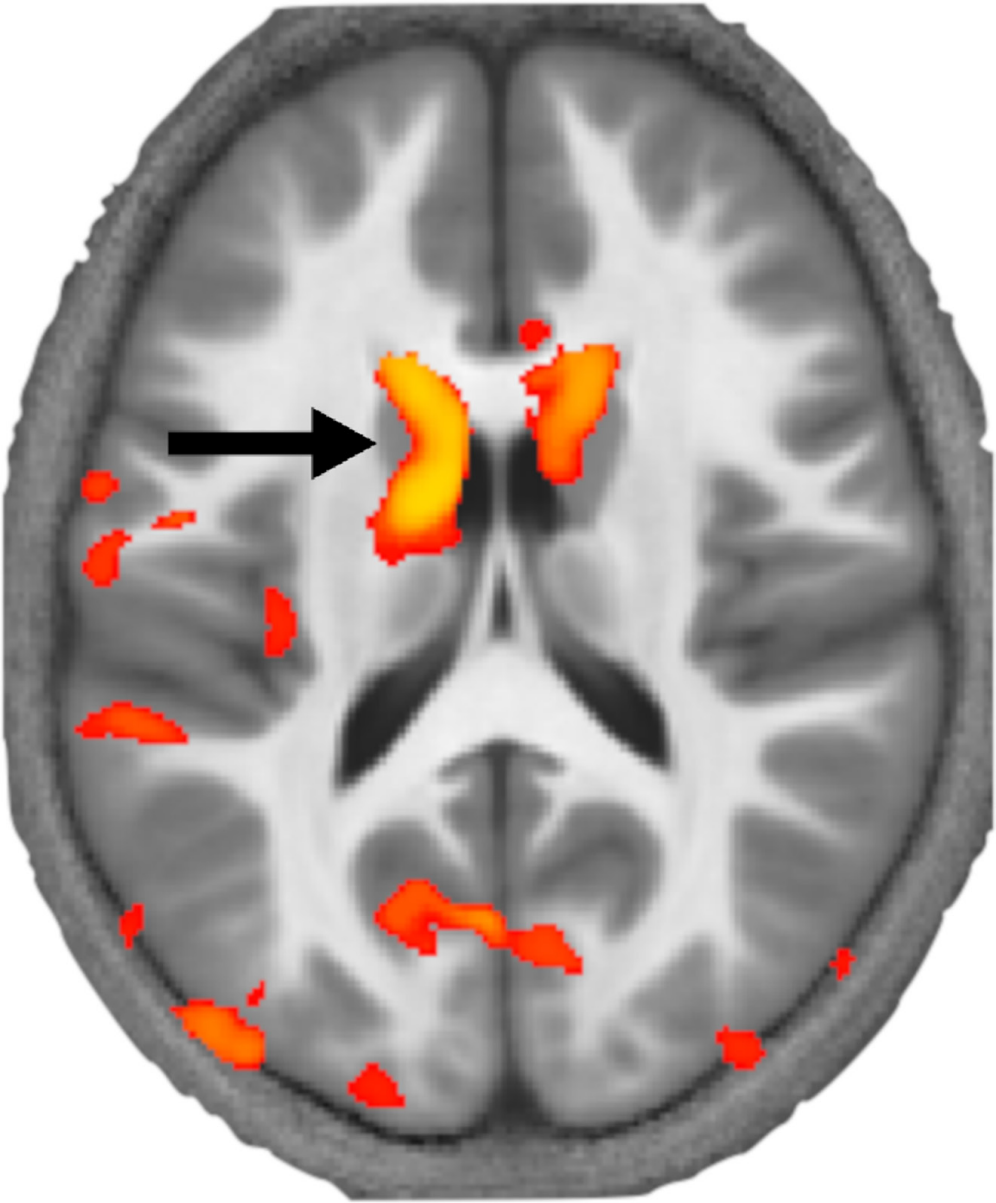
How levels of myelin differ in the brains of children with developmental language disorder. Krishnan et al. used a MRI technique called MTsat to image the brains of children with and without developmental language disorder. This image shows a section through the brain at the level of the basal ganglia with a coloured map (orange and yellow) overlaid representing the areas of the brain where myelin levels are reduced. This revealed that myelin levels are particularly low in a part of the striatum called the left caudate (black arrow) in children with developmental language disorder.

Image credit: Adapted from Figure 1 of Krishnan et al., 2022

Further work is needed to see whether the anatomical differences discovered by Krishnan et al. and others cause DLD: in an association study like this, it is equally possible that DLD is responsible for the observed brain changes. In addition, it remains to be seen whether the molecules responsible for the reduced levels of myelin found in the basal ganglia and Broca’s area are coded by genes that have already been implicated in DLD (Plug et al., 2021).

In the future, brain imaging could be used to inform the diagnosis and characterisation of DLD. However, we are a long way off from this being a routine procedure for individuals, as there was overlap between the brain myelin measurements in children with and without DLD. Nevertheless, the work by Krishnan et al. sheds new light on possible explanations for DLD that may help clinicians diagnose the condition earlier and identify new treatment strategies.
